# Quarantine Conference

**Published:** 1903-02

**Authors:** 


Quarantine Conference.—On the call of State Health Officer Tabor, of Texas, a meeting of representatives of the State Board of Health of Alabama, Mississippi and Texas was held in Galveston January 26 and 27, ult. The object of the conference was to adopt uniform quarantine rules, so that in matters of commerce no discriminations could be made for or against any Southern port; vessels should be treated alike at all. Florida was not represented, because, it is said, the quarantine laws of that State are now controlled by the U. S. Marine Hospital Service. A set of regulations was adopted that is satisfactory to all the authorities represented. They were published in full in the *Galveston News* of January 27th.

In speaking of the result of the meeting, Mr. W. H. Gaines, secretary of the Galveston Maritime Association, said:

"I do not think the report, as adopted, could be improved upon in any respect. I think it covers everything that is necessary, and it has been thoroughly gone over by the best quarantine physicians in the Southern States. I think they have covered the ground in every essential. It puts Galveston on an equal footing with Mobile and New Orleans. It certainly gives us the same advantages they possess and we should share an equal amount of the fruit business. Dr. Tabor deserves a great deal of credit for carrying this matter to a successful issue. He has been working upon it for months, but has never succeeded in getting it through until he arranged with all of the executive officers to meet him. He is a sincere friend of Galveston, and is conscientious in his work and wants to do what is right towards all concerned."

---

The quarantine officers and inspectors under the new administration are: At Galveston, Dr. E. F. McClendon, reappointed; at Sabine Pass, Dr. B. U. Sims, reappointed; at Velasco, Dr. B. H. Carleton, reappointed; at Pass Cavallo, Dr. T. J. McFarland, reappointed; at Brownsville, Dr. A. S. Wolff, reappointed; at Laredo, Dr. J. M. McKnight, reappointed; at El Paso, Dr. A. L. Justice, of El Paso, vice Dr. H. E. Stevenson; at Aransas Pass,

Dr. J. Hicks Florence, of Dallas, vice Dr. W. E. Pugh; at Eagle Pass, Dr. I. H. Brewton, of Del Rio, vice Dr. Malone Duggan.

---

Dr. Fred Terrell is now mayor of San Antonio, and ex-Mayor Marshall Hicks is in the Senate. Both are friends and advocates of sanitation.

